# Sex differences in hepatocellular carcinoma indicated BEX4 as a potential target to improve efficacy of lenvatinib plus immune checkpoint inhibitors

**DOI:** 10.7150/jca.73051

**Published:** 2022-09-06

**Authors:** Lu Liu, Kangkang Yu, Chong Huang, Meisi Huo, Xiaoqi Li, Ruiqi Yin, Chuanmiao Liu, Lu Lu, Huaping Sun, Jubo Zhang

**Affiliations:** 1 Department of Infectious Diseases, Shanghai Key Laboratory of Infectious Diseases and Biosafety Emergency Response, National Medical Center for Infectious Diseases, Huashan Hospital, Fudan University, Shanghai 200040, China.; 2 Department of Infectious Diseases, The First Affiliated Hospital of Bengbu Medical College, Bengbu 233000, China.; 3 Department of General Surgery, Huashan Hospital, Cancer Metastasis Institute, Fudan University, Shanghai 200040, China.; 4 Department of Radiology, Huashan Hospital, Fudan University, Shanghai 200040, China.

**Keywords:** hepatocellular carcinoma, sex difference, immunity, metabolism, therapy

## Abstract

**Background:** Hepatocellular carcinoma (HCC) is the most common form of liver cancer, and significant sex disparities have been observed in HCC. We aim to explore the potential sex-biased mechanisms involved in hepatocarcinogenesis.

**Methods:** Based on TCGA data, we compared clinical features, genetic alterations, and immune cell infiltrations between male and female HCC patients. In addition, we performed sex-based differential expression analysis and functional enrichment analysis. Finally, GSE64041 dataset and another HCC cohort were engaged to validate our findings.

**Results:** Significant differences of genetic alterations and TME were observed between male and female HCC patients. Enhanced metabolism of lipids was associated with hepatocarcinogenesis in men, while ECM-organization-related pathways were correlated to HCC development in women. *BEX4* was upregulated in female but downregulated in male HCC patients, and was positively correlated with immune checkpoint molecules and infiltrated immune cell. These findings were further validated in dataset GSE64041 and our HCC cohort. More importantly, a negative correlation was found between *BEX4* expression and lenvatinib sensitivity.

**Conclusion**: Distinct biological processes were involved in sex-biased tumorigenesis of HCC. *BEX4* can be targeted to improve the efficacy of lenvatinib plus immune checkpoint inhibitors.

## Introduction

Liver cancer is one of the most common cancers and the third leading cause of cancer-related deaths worldwide, with hepatocellular carcinoma (HCC) as the most common type 1, 2. According to the latest global cancer statistics, liver cancer accounts for 4.7% of the 19.3 million new cases and 8.3% of the 9.9 million new deaths globally 1. Specifically, men account for 70.1% of new cases and 70.3% of new deaths 1, significantly higher incidence and mortality in males reflect that HCC has sex disparities, which was considered as an effect dependent on sex hormones 3, 4. Consistent with this, it has been reported that estrogen could inhibit secretion of interleukin-6, a multifunctional cytokine that causes inflammation, thus risk of inflammation-induced liver cancer in women was reduced 5. In addition, Foxa1/2 and their targets have been demonstrated as central players in sexual dimorphism of HCC by interacting with estrogen receptor 3. However, the comprehensive mechanisms of sexual dimorphism in hepatocarcinogenesis remains not fully understood.

Arranged in tandem on chromosome X, Brain-expressed X-linked 4 (*BEX4*) is a member of BEX family which was involved in different signaling pathways and played important roles in physiological and pathophysiological conditions 6, 7. BEX4-mediated YAP/TAZ activation was reported to promote the tumor growth and radioresistance in Glioblastoma 8. In addition, BEX4 was implicated in oncogenic microtubule hyperacetylation by interacting with sirtuin 2 9. On the contrary, BEX4 may act as a tumor suppressor in oral squamous cell carcinoma and ovarian cancer 10, 11. Still, the function of BEX4 in HCC remains obscure to date.

In this study, based on TCGA data, we compared clinical features of HCC between male and female patients, and evaluated the differences of genetic alterations and immune cell infiltrations between male and female patients. In addition, we performed differential expression analysis and enrichment analysis to reveal potential biological processes and pathways that are implicated in sex-biased hepatocarcinogenesis. Finally, we focused on *BEX4*, the unique gene upregulated in female but downregulated in male HCC patients. We found *BEX4* expression positively correlated with immune cell infiltration as well as immune checkpoint molecule expression. These results were further validated in dataset GSE64041. Our findings provided new insight into sex differences in tumorigenesis of HCC and were helpful to the development of sex-based therapeutic strategies.

## Materials and methods

### Data download and analysis

RNA-seq, genetic alteration, and clinicopathological data of HCC patients were downloaded from cBioPortal (https://www.cbioportal.org) 12. The enrollment criteria for this study were as follows: First, all patients were diagnosed as having HCC by pathology. Second, BEX4 mRNA expression data and clinical features including gender, age, race, TNM stage, tumor grade, clinical stage were available. Third, informed consent was acquired from all patients by the TCGA Research Network. A total of 371 patients who met these criteria were enrolled. Differential expression analysis was performed using R package LIMMA with online tool BART 13, genes with |fold change|≥1.5 and adjusted p<0.05 were considered to be differentially expressed genes. Immune cell infiltration levels inferred by CIBERSORT algorithm 14 were downloaded from GDC database (https://gdc.cancer.gov/). GSE64041 dataset was downloaded from GEO database (https://www.ncbi.nlm.nih.gov/geo/).

### CVCDAP database analysis

Cancer virtual cohort discovery analysis platform (CVCDAP, https://omics.bjcancer.org/cvcdap/home.do) is an integrated web tool for molecular and clinical analysis of cancer cohorts 15. CVCDAP was engaged to analyze and visualize the top 5 most frequently mutated genes in HCC.

### GEPIA database analysis

Gene expression profiling interactive analysis (GEPIA2.0, http://gepia2.cancer-pku.cn/#index) is a web-based platform which allow investigators to analyze RNA sequencing expression data deposited in TCGA and GTEx 16. GEPIA was used to perform correlation analysis between BEX4 and immune checkpoint-related molecules.

### Enrichment analysis

Gene set enrichment analysis (GSEA) 17 was applied to identify functions and pathways associated with hepatocarcinogenesis. The differentially expressed genes were subjected to Metascape (https://metascape.org/gp/index.html) to perform network enrichment analysis 18. Protein-protein interaction (PPI) network was analyzed by STRING database (https://string-db.org/) 19 and visualized by Cytoscape (version 3.7.2) 20.

### TISIDB database analysis

TISIDB (http://cis.hku.hk/TISIDB/index.php) is a web portal for tumor and immune system interactions 21. We employed TISIDB to explore the distributions of *BEX4* expression across immune/molecular subtypes of HCC. In addition, correlations between *BEX4* expression and clinical stage/tumor grade of HCC were also evaluated.

### TIMER database analysis

TIMER (https://cistrome.shinyapps.io/timer/) is a web resource for systematical assessment of the clinical impact of tumor-infiltrating immune cells in various human cancers 22, 23. TIMER was engaged here to estimate the correlation between *BEX4* expression and infiltration levels of immune cells. Besides, correlations between expression of *BEX4* and immune checkpoint molecules as well as immune cell markers were also assessed.

### GSCA database analysis

Gene set cancer analysis (GSCA, http://bioinfo.life.hust.edu.cn/GSCA/#/) is an integrated online resource for genomic and immunogenomic gene set cancer analysis 24. GSCA also integrated small molecule drugs from the Cancer Therapeutics Response Portal. GSCA was engaged to evaluate correlation between *BEX4* expression and small molecule drug sensitivity.

### RT-qPCR

Validation study utilized 40 paired human HCC samples collected in previous studies 25. RT-qPCR reactions were performed using SYBR premix Ex Taq™ II kit (TAKARA BIO INC.) according to the manufacturer's instructions. Specific primers for each gene were listed as followed:

GAPDH forward: TGCGAGTACTCAACACCAACA; GAPDH reverse: GCATATATTCGGCCCACA; BEX4 forward: AAAGAGGAACTAGCGGCAAAC; BEX4 reverse: CCAAATGGCGGGATTCTTCTTC.

### Immunohistochemistry (IHC)

For validation cohort, BEX4 expression was detected by IHC. IHC staining was carried out using an anti-BEX4 antibody (rabbit polyclonal, Sinobiological) at 1:50 dilution as previously described 25. BEX4 intensity score was qualified as no staining (score 0), weak (score 1), medium (score 2), and strong (score 3). The percentage of positive cells was divided into 4 scores: 1 = <25%, 2 = 25% to 50%, 3 = 50% to 75%, 4= >75%. The final IHC scores = intensity score × percentage score.

### Flow Cytometry

32 HCC tissue were collected for flow cytometry analysis in previous studies. The tissue digestion, grinding and flow cytometry staining were performed as previously described 26. CD4^+^ T cells, CD8^+^ T cells, B cells and macrophages were determined as CD45^+^CD3^+^CD4^+^CD8^-^, CD45^+^CD3^+^CD4^-^CD8^+^, CD45^+^CD3^-^CD19^+^ and CD45^+^CD14^+^CD68^+^, respectively.

### Statistical analysis

Statistical difference was analyzed using t-test between two groups. Chi-square and Fisher tests were performed for categorical variables. Spearman correlation was used for correlation analysis. Statistical analysis for figures generated from online databases was calculated automatically. p value <0.05 was considered statistically significant.

## Results

### Clinicopathological characteristics

**Figure 1:**
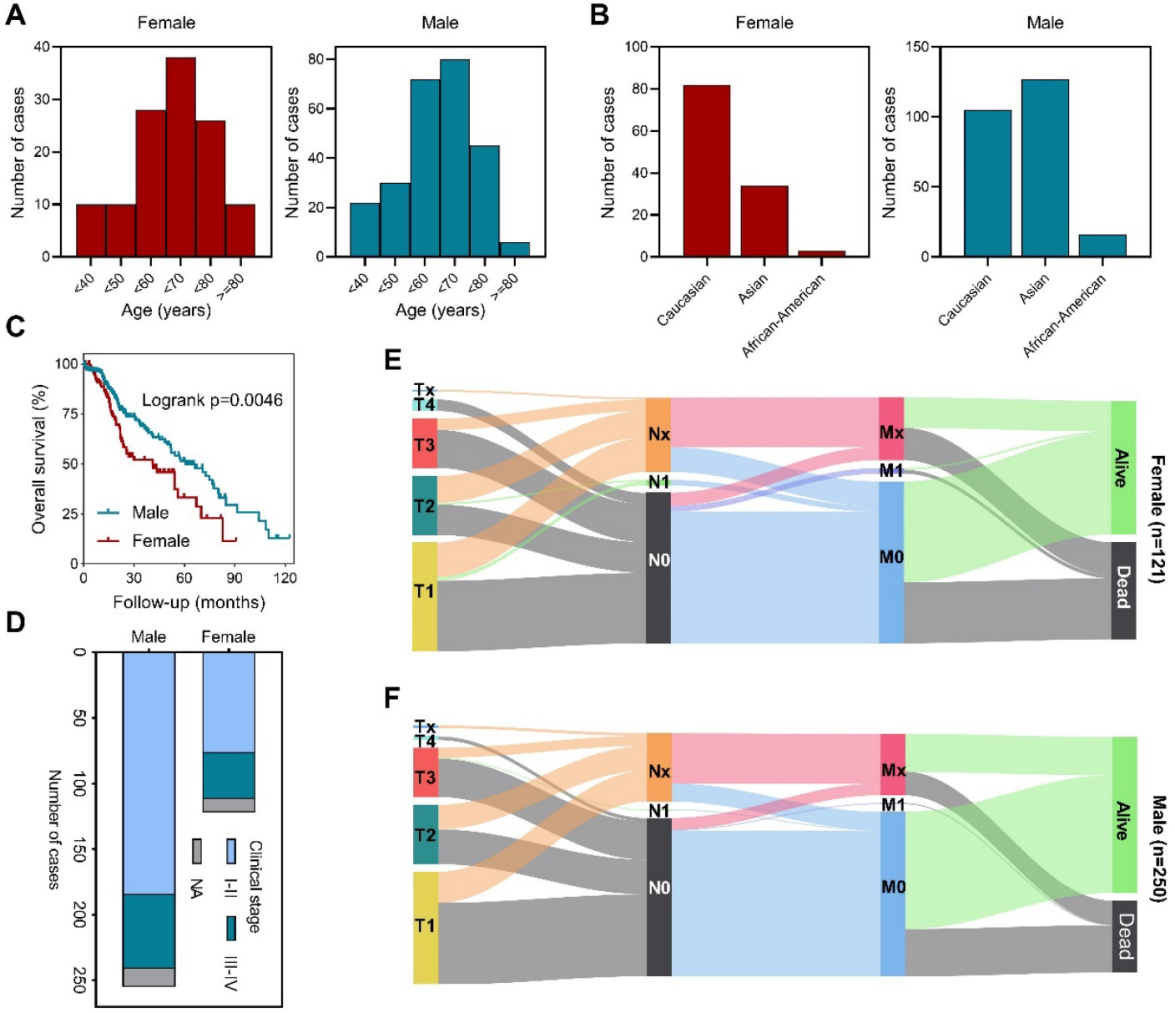
** Sex-based clinicopathological analysis of HCC patients. (A)** Age distribution among male and female patients. **(B)** Race distribution among male and female patients. **(C)** Sex-based survival analysis. **(D)** Clinical stage distribution among male and female patients. **(E)** Distribution of TNM stages in female patients. **(F)** Distribution of TNM stages in male patients.

We first analyzed the age distribution of male and female patients and found that most patients were aged between fifty to eighty in both groups (Figure 1A). Exploration based on race showed that female patients are predominantly Caucasian, while male patients are mostly Asian and Caucasian (Figure 1B). Survival analysis revealed that male patients had longer overall survival (Figure 1C). Further investigation verified that stage I-II patients were more common in male (Figure 1D). Detailed primary tumor, lymph node, metastasis (TNM stage) distribution analysis demonstrated that male patients had lower T stage, less lymph node involvement and distal metastasis than female patients (Figures 1E,F). These data showed that in TCGA cohort, there was no significant difference in the age distribution of HCC patients by gender, but more male patients were in early stages of the disease. Clinicopathologic characteristics of enrolled cohort and the correlation between clinicopathologic characteristics and BEX4 were further summarized (Table ​S1 and Table S2).

### Sex-differential genetic alterations

**Figure 2:**
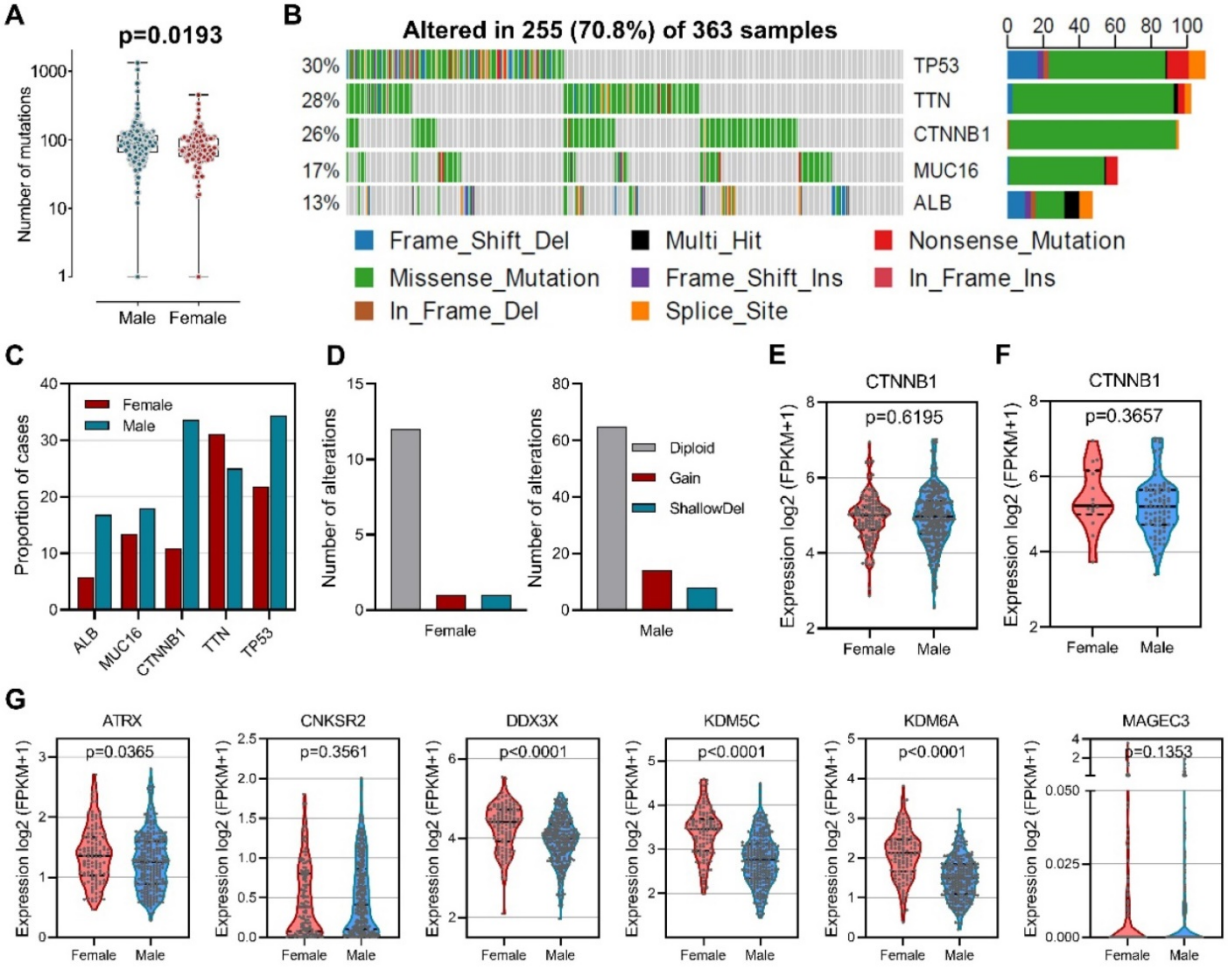
** Sex-based genetic alteration analysis. (A)** Comparison of total mutation numbers between male and female patients. **(B)** Mutation plots of the top 5 most frequently mutated genes in HCC. **(C)** Mutation frequencies of the top 5 mutated genes in male and female patients. **(D)** Copy number alteration of *CTNNB1* in male and female patients. **(E)** Comparison of *CTNNB1* expression in male and female patients. **(F)** Comparison of *CTNNB1* expression in *CTNNB1* mutated male and female patients. **(G)** Expression comparison of tumor suppressor genes that escape from X-inactivation.

To explore the difference of genetic alterations between males and females in HCC, we determined mutated genes using cBioPortal database. Our results showed that male HCC patients had higher numbers of mutations than female HCC patients (Figure 2A). Among the 363 samples, the top five most frequently mutated genes in HCC were *TP53* (30%), *TTN* (28%), *CTNNB1* (26%), *MUC16* (17%), and *ALB* (13%) (Figure 2B). Mutation frequencies were higher in male patients than in female patients for *TP53* (34.4% versus 21.8%), *CTNNB1* (33.6% versus 10.9%), *MUC16* (18.0% versus 13.4%), and *ALB* (16.8% versus 5.8%), while mutation frequency of *TTN* (25.0% versus 31.1%) was lower (Figure 2C). *CTNNB1* mutation has been identified as a sex-biased driver mutation in HCC 27-29, we then analyzed copy number alteration of *CTNNB1* and found increased copy number gain in male-derived samples (Figure 2D). However, no differences in *CTNNB1* transcript levels were observed between male and female in both total patients (Figure 2E) and *CTNNB1* mutated patients (Figure 2F). In addition, a group of tumor suppressors that escape from X-inactivation have been implicated in cancer sex bias 30, our analysis did reveal higher expression of *ATRX*, *DDX3X*, *KDM5C* and *KDM6A* in female than male patients (Figure 2G). These results indicated that gene alterations are associated with sex-biased carcinogenesis of HCC.

### Sex-differential immune alterations

**Figure 3:**
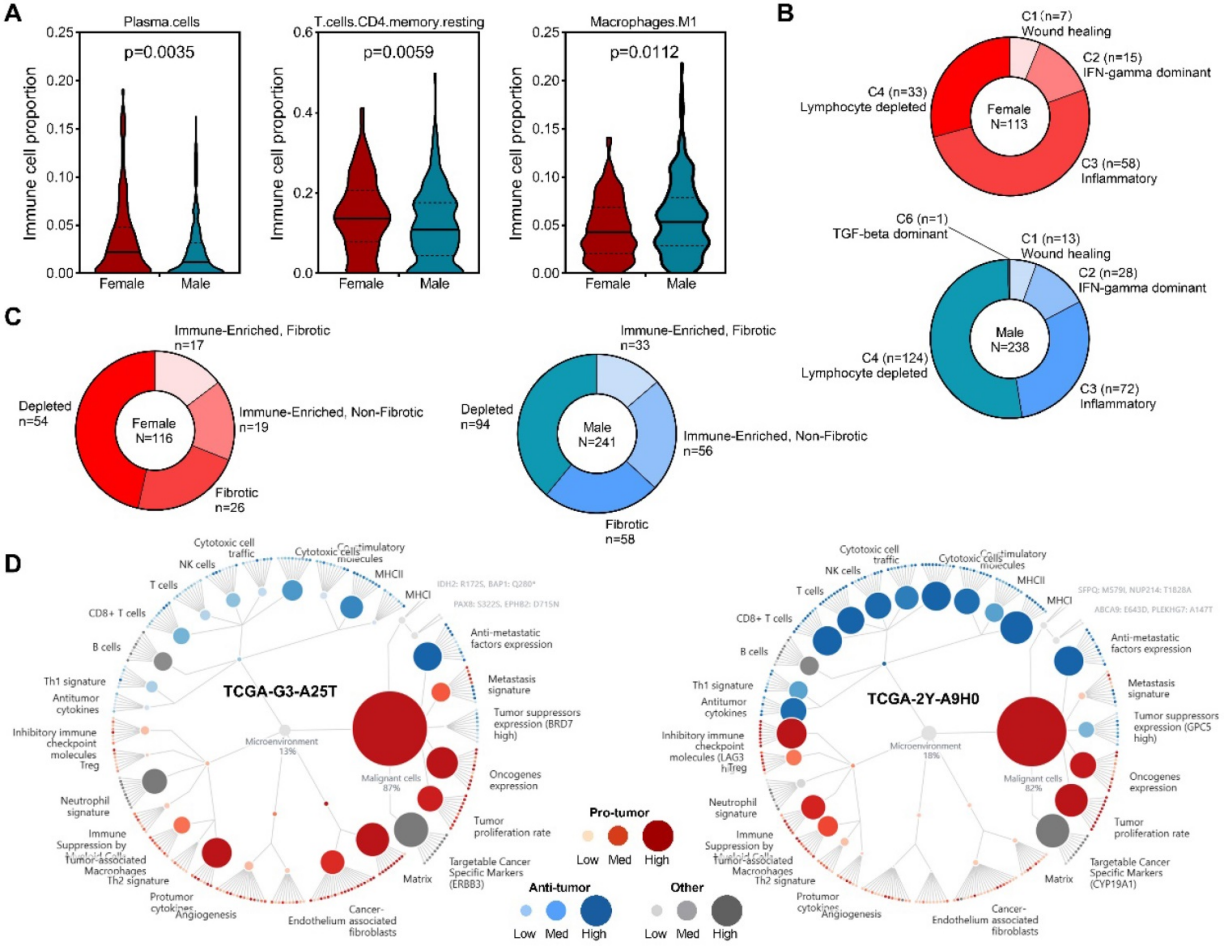
** Sex-based immune alteration analysis. (A)** Immune cells with different infiltration levels between male and female patients. **(B)** Immune cluster distribution among male and female patients. **(C)** TME subtype distribution among male and female patients. **(D)** Representative TME subtypes, Depleted (left) and Immune-Enriched, Non-Fibrotic 37.

To get insight into sex differences of immune cell infiltration in HCC, RNA-seq data from TCGA project was subjected to CIBERSORT to infer immune cell populations. Overview of tumor-infiltrating immune cell populations arrange by sex was presented (Figure S1). Our results demonstrated that compared with male patients, female patients had higher infiltration levels of plasma cells and CD4 memory resting T cells, but lower levels of M1 macrophages (Figure 3A). A previous study identified six immune clusters in human malignancies that are associated with tumor microenvironment (TME) and patient's prognosis 31. We found that in female patients, inflammatory subtype was the dominant cluster, followed by lymphocyte depleted subtype (Figure 3B). In male patients, however, most patients belonged to lymphocyte depleted subtype, while inflammatory subtype was the second largest cluster (Figure 3B). Recently, a study identified four TME subtypes (Immune-enriched, fibrotic, IE/F; Immune-enriched, non-fibrotic, IE; Fibrotic, F; Depleted, D) that correlated with patient response to immunotherapy 32, our analysis revealed increased immune-enriched, non-fibrotic subtype and decreased depleted subtype in male patients (Figure 3C), and representative molecular function portraits of HCC with TME subtype IE and D were displayed. These findings suggested sex-biased TME in HCC.

### Sex-based gene set enrichment analysis

**Figure 4:**
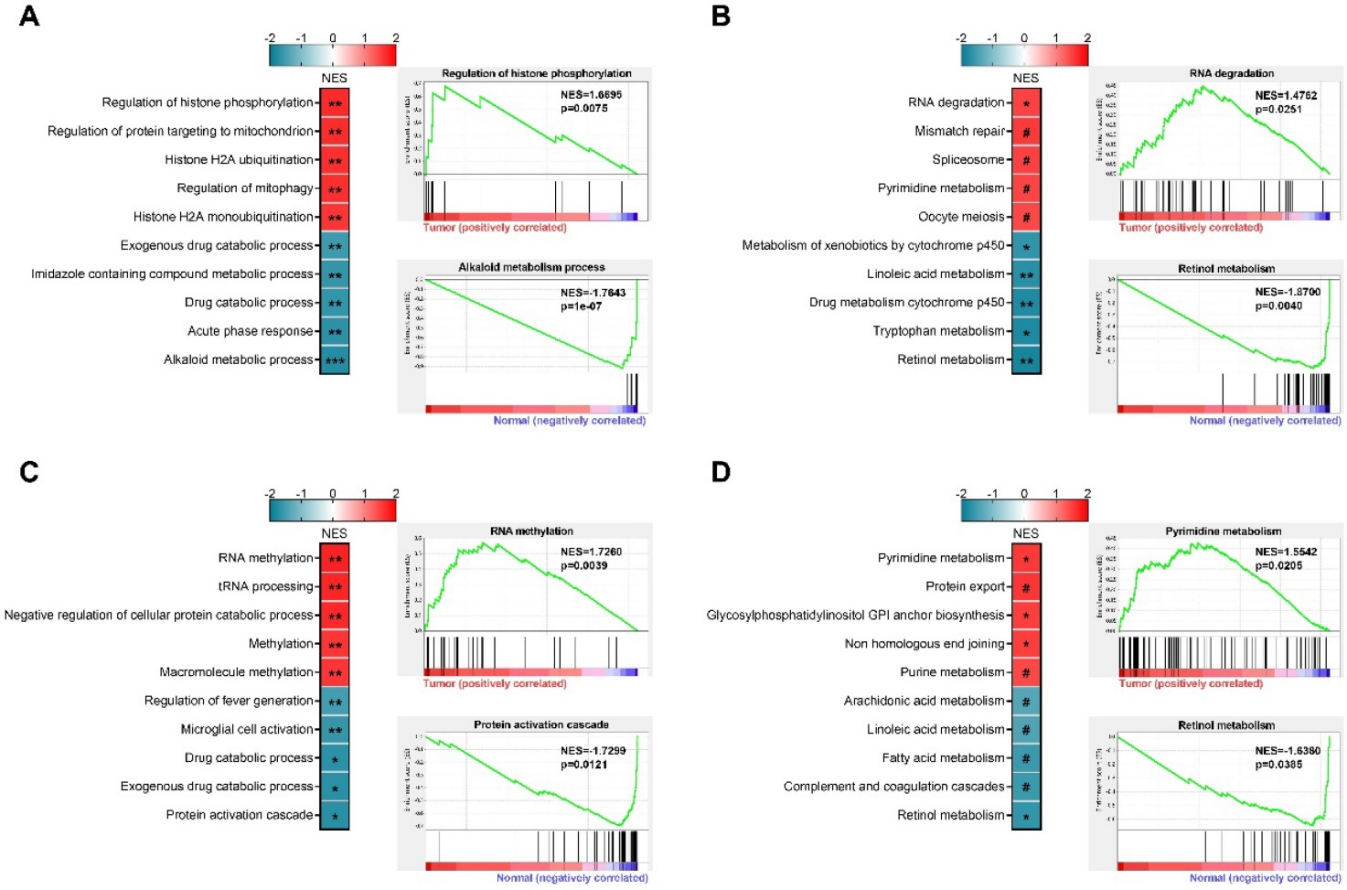
** Sex-based gene set enrichment analysis. (A)** Top 5 positively and negatively enriched GO terms in female patients, representative enrichment plots were presented. **(B)** Top 5 positively and negatively enriched KEGG pathways in female patients, representative enrichment plots were presented. **(C)** Top 5 positively and negatively enriched GO terms in male patients, representative enrichment plots were presented. **(D)** Top 5 positively and negatively enriched KEGG pathways in male patients, representative enrichment plots were presented. NES, normalized enrichment score; ***p<0.001, **p<0.01, *p<0.05, #p>0.05.

Based on paired RNA-seq data of male and female patients from TCGA project, we performed GSEA analysis and the top 5 positively and negatively enriched gene sets were presented (Figures 4A-D). In female patients, Alkaloid metabolic process, Acute phase response, Drug catabolic process, Imidazole containing compound metabolic process, and Exogenous drug catabolic process were the top 5 negatively enriched GO biological processes (Figure 4A), while Regulation of histone phosphorylation, Regulation of protein targeting to mitochondrion, Histone H2A ubiquitination, Regulation of mitophagy, and Histone H2A monoubiquitination were the top 5 positively enriched GO biological processes (Figure 4A). The top 5 negatively enriched KEGG pathways were Retinol metabolism, Tryptophan metabolism, Drug metabolism cytochrome P450, Linoleic acid metabolism, and Metabolism of xenobiotics by cytochrome P450 (Figure 4B). RNA degradation, Mismatch repair, Spliceosome, Pyrimidine metabolism, and Oocyte meiosis were the top 5 positively enriched KEGG pathways (Figure 4B). In male patients, the top 5 negatively enriched GO biological processes were Regulation of fever generation, Microglial cell activation, Drug catabolic process, Exogenous drug catabolic process, and Protein activation cascade (Figure 4C), while RNA methylation, tRNA processing, Negative regulation of cellular protein catabolic process, Methylation, and Macromolecule methylation were the top 5 positively enriched GO biological processes (Figure 4C). The top 5 negatively enriched KEGG pathways were Retinol metabolism, Complement and coagulation cascades, Fatty acid metabolism, Arachidonic acid metabolism, and Linoleic acid metabolism (Figure 4D), while the top 5 positively enriched KEGG pathways were Pyrimidine metabolism, Protein export, Glycosylphosphatidylinositol anchor biosynthesis, Non-homologous end joining, and Purine metabolism (Figure 4D. These findings highlighted sex-biased metabolism reprogramming in hepatocarcinogenesis, which may serve as regions of interest for further exploration.

### Functional enrichment analysis of DEGs between male and female patients

**Figure 5:**
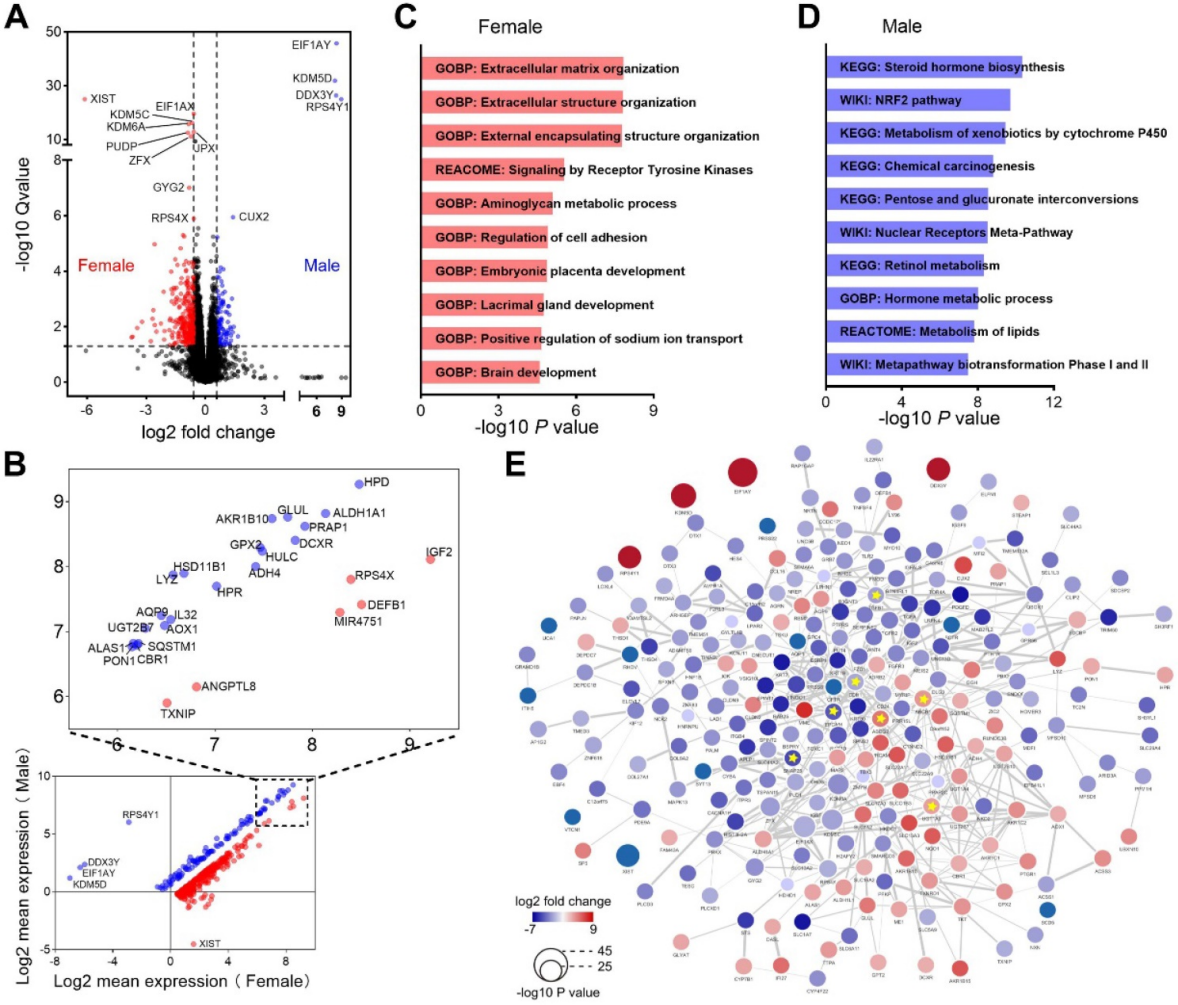
** Functional enrichment analysis of DEGs between male and female patients. (A)** Heatmap shows DEGs between male and female patients. **(B)** Average expression of the DEGs in male and female patients. **(C)** Top 10 pathways enriched in female patients. **(D)** Top 10 pathways enriched in male patients. **(E)** PPI interaction network of the DEGs. Hub genes were marked with yellow star, edge thickness indicates interaction score (range from 0.400 to 0.996).

We next evaluated genes differentially expressed between male and female HCC patients. The comparison revealed that 110 genes were upregulated, while 279 genes were downregulated in male patients (Figure 5A). Most of these dysregulated genes possessed higher expression levels both in male and female patients, and the top expressed genes were labeled (Figure 5B). Enrichment analysis demonstrated that genes upregulated in female patients were mainly enriched in extracellular matrix organization and receptor tyrosine kinase signaling (Figure 5C). In contrast, genes upregulated in male patients were mainly involved in metabolism of lipids and carbohydrates (Figure 5D). We also performed PPI network analysis and identified 7 highly connected hub genes, including 4 genes (*CDH1*, *EPCAM*, *SNAP25* and *TGFB1*) upregulated in female and 3 genes (*ABCB1*, *ABCG2* and *UGT1A9*) upregulated in male (Figure 5E). *CDH1*, *EPCAM* and *TGFB1* play important roles in epithelial-mesenchymal transition (EMT), a process associated with tumorigenesis 33.* ABCB1*, *ABCG2* and *UGT1A9* were critical molecules that mediate chemotherapeutic drug resistance 34-36. These results reflected that different genes and pathways were implicated in the development and progression of HCC in men and women.

### Functional enrichment analysis of sex-specific dysregulated genes

**Figure 6:**
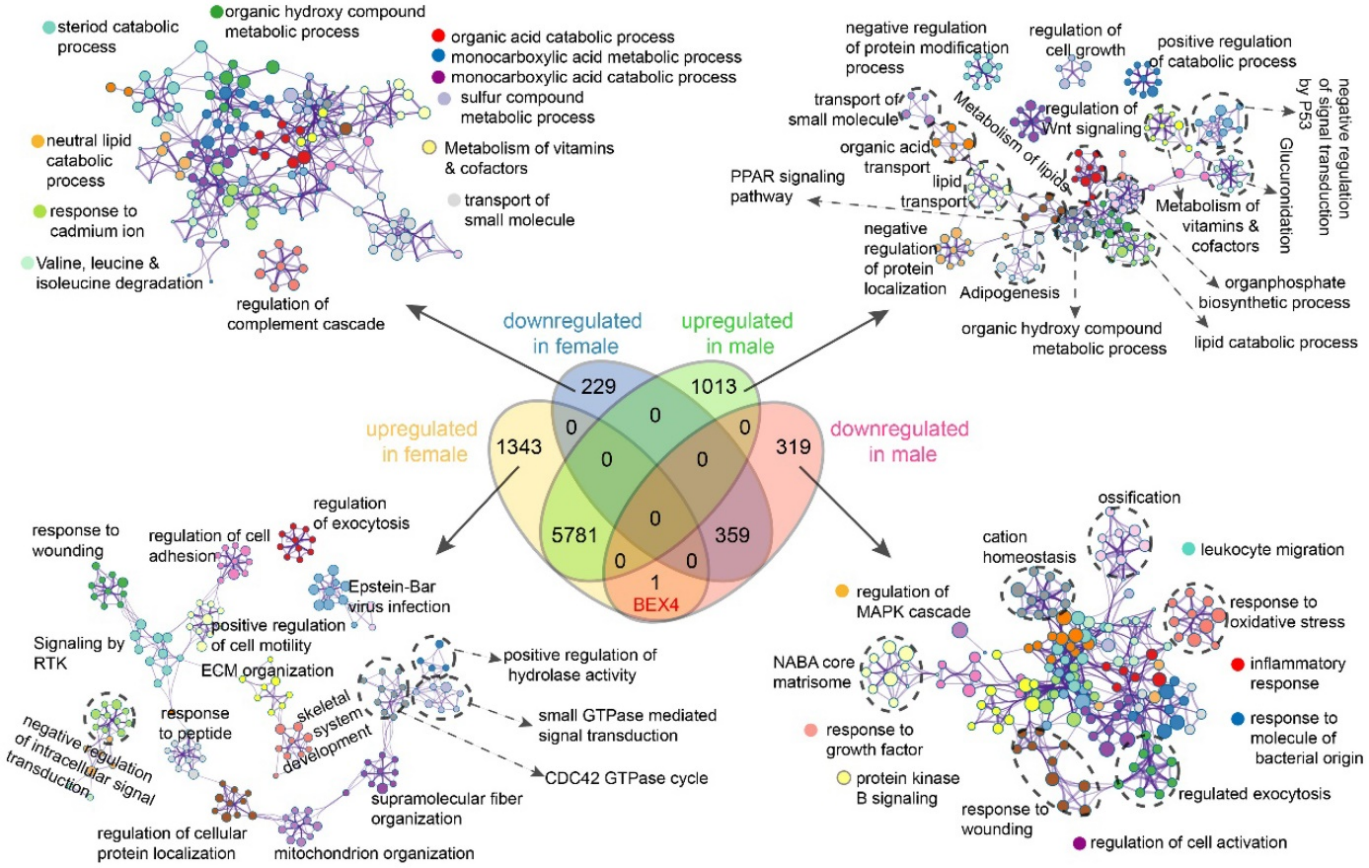
** Sex-based functional enrichment analysis.** Enrichment pathway networks of genes exclusively downregulated in female, upregulated in female, downregulated in male, and upregulated in male.

We then performed differential expression analysis based on these paired RNA-seq data and found that dysregulated genes in female patients were significantly fewer than that in male patients (Figure S2). Next, functional enrichment analyses of genes exclusively up-/down-regulated in male or female patients were implemented (Figure 6, Figure S3). Our results showed that genes exclusively downregulated in female patients were enriched in terms related to metabolic or catabolic processes of acids, lipid, steroid, and vitamins (Figure 6). Genes exclusively upregulated in female patients were mainly enriched in terms associated with signal transduction, ECM organization, mitochondrion organization, and protein localization (Figure 6). While in male patients, upregulated genes were enriched in terms related to signal transduction, material transport, protein modification/localization, metabolic and catabolic and biosynthetic processes, especially lipid metabolism related processes and signal transduction by P53 (Figure 6). In contrast, downregulated genes were mainly enriched in immune related processes including leukocyte migration, inflammatory response, response to molecule of bacterial origin, and cell activation (Figure 6). These results further emphasized the differences between male and female in hepatocarcinogenesis.

### Potential role of *BEX4* in HCC development

**Figure 7:**
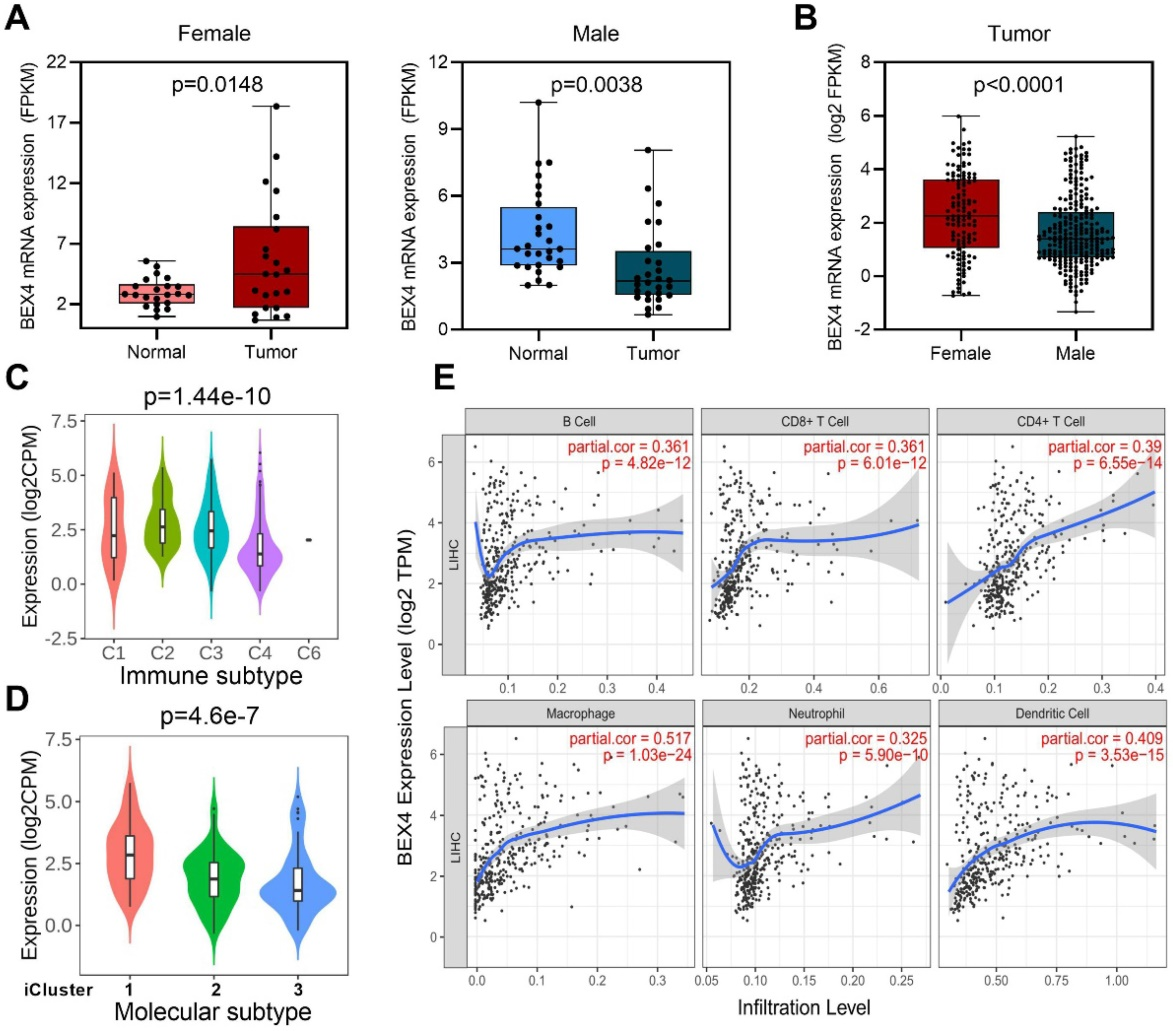
** Function exploration of *BEX4* in HCC. (A)** Comparison of *BEX4* expression between paired normal and tumor samples in male and female patients. **(B)** Comparison of *BEX4* expression between male and female patients. **(C)** Comparison of *BEX4* expression across six immune subtypes. **(D)** Comparison of *BEX4* expression across three molecular subtypes. **(E)** Correlation between *BEX4* expression and immune cell infiltration.

We then focused on Brain-expressed X-linked 4 (*BEX4*), the unique gene which upregulated in female but downregulated in male (Figure 6, Figure 7A). Expression analysis revealed that female HCC patients possessed higher *BEX4* transcriptional levels than male patients (Figure 7B). Although no strong correlation was observed between *BEX4* mRNA level and tumor grade (Figure S4A) or disease stage (Figure S4B), significantly differential expression of *BEX4* was observed across immune and molecular subtypes of HCC (Figures 7C-D). Moreover, we evaluated the correlation between *BEX4* expression and immune cell infiltration*.* The results revealed a robust positive association between mRNA levels of *BEX4* and infiltrating immune cells, especially macrophage (Figure 7E). Similarly, strong positive correlations were found between immune checkpoint molecules and *BEX4* expression (Figure S4C). Further validated in GSE64041 (Figure S5), these findings suggested that *BEX4* can regulate anti-tumor immunity by modulating TME. Interestingly, data mined from the Cancer Therapeutics Response Portal (CTRP) 37 via GSCA indicate that *BEX4* expression negatively correlates with sensitivity to multiple receptor tyrosine kinases (lenvatinib, axitinib, and AZD4547) (Figure S4D). Given that lenvatinib plus anti-PD-1 antibody can enhance antitumor activity in HCC by decreasing tumor-associated macrophage 38, 39, targeting *BEX4* may further improve the efficacy of the combination therapy.

### Validation analyses using our HCC cohort

**Figure 8:**
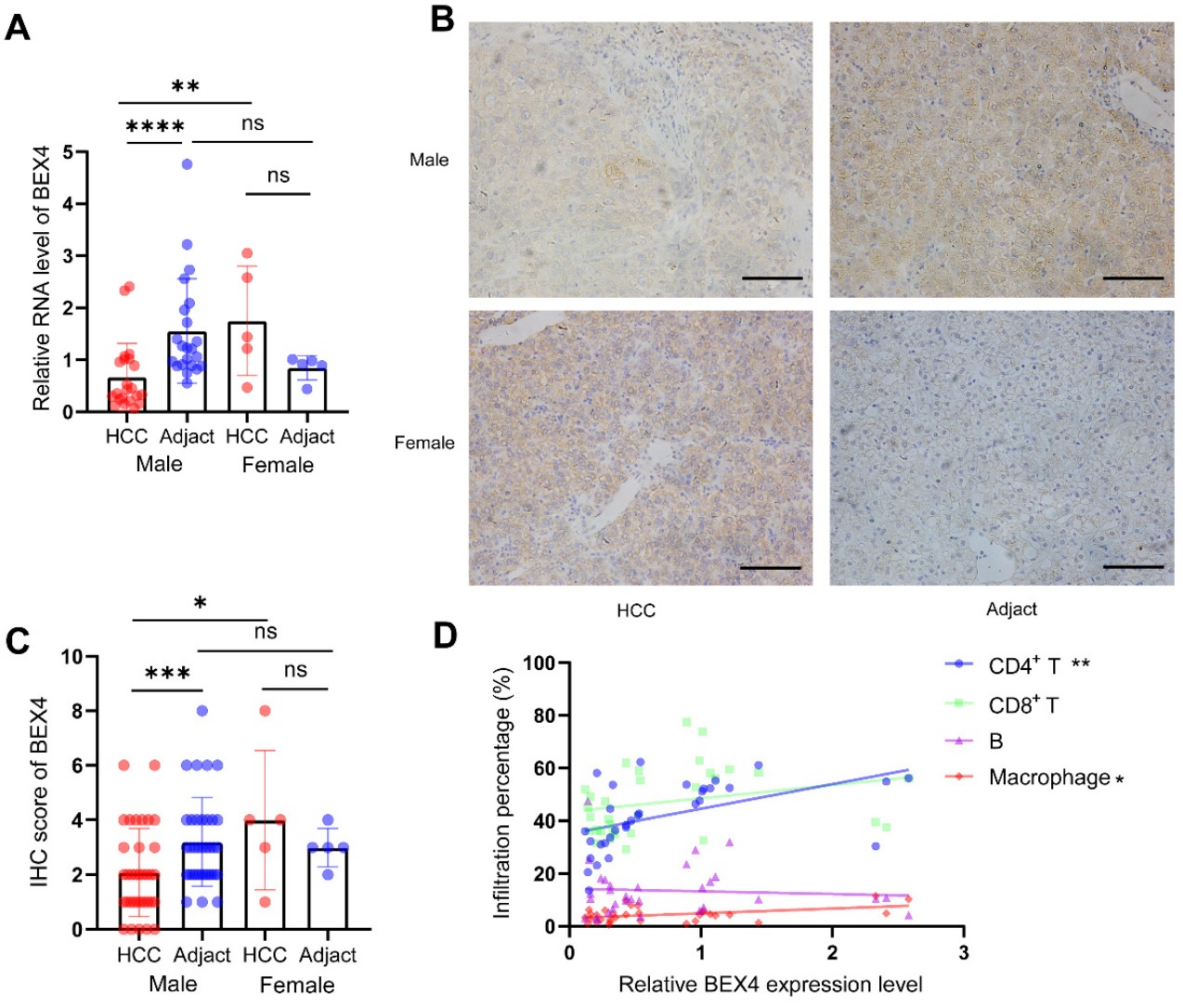
** Validation analyses using our HCC cohort. (A)** RT-qPCR validation of BEX4 expression. **(B,C)** IHC validation of BEX4 expression. Scale bar=200 um. **(D)** Correlations between CD4^+^ T cells, CD8^+^ T cells, B cells, macrophages and BEX4 level. ****P<0.0001, ***P<0.001, **P<0.01, *P<0.05.

To validate our findings based on bioinformatic analyses, 40 HCC tissues and their adjacent tissues were collected. RT-qPCR suggested female HCC patients had higher BEX4 expression than male patients. The level of BEX4 in the cancerous tissue was significantly reduced compared with the paracarcinoma tissue in male patients. However, probably due to the small sample size, no statistical significance was reached in female patients (Figure 8A). BEX4 protein level was measured by Immunohistochemistry, similar results were found, which were consistent with our RT-qPCR results (Figure 8C). Representative images were shown in Figure 8B. Moreover, to validate the correlation between infiltrated immune cells and BEX4 level, flow cytometry was performed in 32 HCC samples. The results, as shown in Figure 8D, indicated that although no significant correlations were observed in CD8^+^ T cells and B cells group, BEX4 was positively correlated with CD4^+^ T cells (r=0.4914, P<0.01) and macrophages (r=0.4436, P<0.05).

## Discussion

In cancer-related studies, sex as a critical biological variable is often understudied 40-42. It is consensus that there are sex differences in incidence across human cancers, especially HCC 41, 42. HCC is one of the tumors with the worst prognosis, and men show significantly higher incidence than women 3, 43. Although sex hormones have been suggested as major contributors 3, 4, the exact mechanisms remain unclear. A better understanding of the potential impact of sex on hepatocarcinogenesis is of great importance for precision medicine in HCC.

In the current study, we found that most HCC patients were aged between 50 to 80, predominantly Caucasian and Asian. Survival analysis revealed that male patients had better outcome than female patients. Further exploration showed that, in TCGA dataset, male HCC patients are mostly in the early stage of the disease.

Cancer has long been regarded as a disease of the genome and gene mutation has been implicated in carcinogenesis 44, 45, knowledge about sex-specific genetic and genome-wide influences in cancer has been reviewed elsewhere 45. We here compared mutation numbers between male patients and female patients and found that male patients had significantly higher mutation numbers than female patients. Consistent with this, the top 5 most mutated genes in HCC showed different mutation frequency in male and female patients. Higher mutation frequencies of *TP53*, *CTNNB1*, *MUC16*, and *ALB* were observed in male patients, while higher mutation frequency of *TTN* was found in female patients. *TTN* is the largest protein in human, and genetic mutation of *TTN* was associated hereditary heart diseases 46. In muscle cells, the product of *TTN* plays an important role in providing connections at individual microfilament level, while in non-muscle cells it contributes to condensation and segregation of chromosome during mitosis 47, 48. Chromosome condensation leads to insulation of transcriptional factors and epigenetic regulators and reduces transcriptional activity, resulting in suppressed gene expression. Epigenetic regulation of gene expression was shown sex-biased pattern 40. Lately, it is reported that solid stress generated by lesion growth can impair infiltration of cancer-specific T cells into lymph node metastases 49. Further investigations are needed to explore whether *TTN* plays roles in sex-specific chromatin regulation and/or solid stress derived from tumor growth. *CTNNB1* mutation is a sex-biased driver mutation in HCC 27, 29, although increased copy number gain was observed in male patients, no significant difference in the transcript levels of *CTNNB1* was detected between male and female, the function of mutated *CTNNB1* therefore warrants further investigation. In addition, higher expression of *ATRX*, *DDX3X*, *KDM5C* and *KDM6A* was observed in female patients, indicating that tumor-suppressor genes that escape from X-inactivation may contribute to HCC sex bias.

Tumors are not simply collections of cancer cells, coupled with stromal cells and extracellular matrix components they established an environment called tumor microenvironment (TME) 32, 50. TME plays a critical role in tumor development and progression, as well as clinical outcomes and response to anti-cancer therapies 50, 51. We found increased infiltration levels of plasma cells and CD4 memory resting T cells in female patients, and M1 macrophages in male patients. Among the six immune clusters associated with TME, female patients are predominantly inflammatory cluster, while male patients are predominantly lymphocyte depleted cluster. Moreover, TME subtype analysis revealed more immune-enriched, non-fibrotic subtype patients in male and more depleted subtype patients in female. These findings highlighted that the TME differs between male and female patients, and taking gender and TME into account will help to guide making precise treatment decision.

Understanding the potential molecular mechanisms of sex-biased cancer biology is vital to improve patient care 27, 52, 53. Comparison between male and female HCC patients uncovered several hub genes and demonstrated distinct signaling pathway activity alteration, especially diminished EMT in men and enhanced anticancer drug resistance in women. In addition, sex-based differential expression analysis revealed that men had more dysregulated genes than women. In female patients, GSEA demonstrated that alkaloid metabolic process was the most negatively enriched biological process, while the most positively enriched biological process was regulation of histone phosphorylation. Besides, retinol metabolism was the most negatively enriched pathway and RNA degradation was the most positively enriched pathway. In male patients, our analysis showed that protein activation cascade and RNA methylation were the most negatively and positively enriched biological processes, respectively. In addition, retinol metabolism and pyrimidine metabolism were the most negatively and positively enriched pathways, respectively. Similarly, functional enrichment analyses demonstrated that genes exclusively up-/down-regulated in male or female patients were involved in almost totally different biological network. In female patients, downregulated genes were mainly involved in various metabolic and catabolic processes, while upregulated genes were chiefly implicated in intracellular signal transduction and extracellular matrix organization-related pathways. In male patients, upregulated genes were enriched in molecule transport as well as adipogenesis-related processes and for downregulated genes, immune response-related processes were the most enriched pathway networks. Sex differences in adipose distribution, adipose tissue and liver substrate metabolism have been well documented, and adipose tissue has been identified as contributor to sex differences in HCC incidence 43, 54. These data improved our understanding of sex-biased molecular mechanisms of HCC.

Brain-expressed X-linked 4 (*BEX4*) has been associated with several cancers, but whether it exerts pro- or anti-cancer effects depending on the tumor type 9-11, 55. Here we found *BEX4* was upregulated in female patients and expression of *BEX4* differed significantly among immune subtypes as well as molecular subtypes of HCC. Moreover, we observed strong positive correlations between transcript levels of *BEX4* and infiltration of various immune cells as well as immune checkpoint molecules. Interestingly, *BEX4* expression was positively correlated with resistance to multiple receptor tyrosine kinases, including lenvatinib. Thus targeting *BEX4* may represent a potential approach to enhance efficacy of lenvatinib plus anti-PD1 antibody combination therapy in HCC.

In summary, our study revealed significant differences of genetic alterations and TME between male and female HCC patients. Enrichment analyses revealed distinct biological processes were involved in sex-biased tumorigenesis of HCC. Enhanced metabolism of lipids was associated hepatocarcinogenesis in men, while ECM-organization-related pathways were correlated to HCC development in women. In addition, *BEX4* was found upregulated in female but downregulated in male HCC patients, and was positively correlated with immune checkpoint molecules and infiltrated immune cell. These results deepened our understanding of sex differences in HCC, and will help to elucidate sex-biased molecular mechanism of tumorigenesis. Further exploration will contribute to the development of novel therapeutic strategies and improve efficacy of lenvatinib plus immune checkpoint blockade combination therapy.

## Supplementary Material

Supplementary figures and tables.

## Abbreviations

HCC: hepatocellular carcinoma
Bex4: brain-expressed x-linked 4
CVCDAP: cancer virtual cohort discovery analysis platform
GEPIA: gene expression profiling interactive analysis
GSEA: gene set enrichment analysis
GSCA: gene set cancer analysis
RT-qPCR: real-time quantitative polymerase chain reaction
IHC: immunohistochemistry
DEGs: differentially expressed genes
TCGA: the cancer genome atlas
KEGG: kyoto encyclopedia of genes and genomes
TME: tumor microenvironment
ECM: extracellular matrix
EMT: epithelial-mesenchymal transition
