# Supplementary material for: Sex differences in hepatocellular carcinoma indicated BEX4 as a potential target to improve efficacy of lenvatinib plus immune checkpoint inhibitors
Source: J Cancer. 2022 Sep 6;13(11):3221–33. doi: 10.7150/jca.73051 (PMC9475366; doi:10.7150/jca.73051)
Supplement: Supplementary file 1 — Supplementary figures and tables. [file jcav13p3221s1.pdf]

Figure S1. Immune cell infiltration analysis. Overview of levels of the 22 tumor-infiltrating immune cells in male and female patients.

Figure S2. Sex-based gene differential expression analysis. Scatter plot shows distribution of DEGs in female and male patients.

Figure S3. Sex-based functional enrichment analysis
(A) Enriched pathways of genes exclusively downregulated in female patients.
(B) Enriched pathways of genes exclusively upregulated in female patients.
(C) Enriched pathways of genes exclusively downregulated in male patients.
(D)Enriched pathways of genes exclusively upregulated in male patients.

Figure S4. Analysis of BEX4 in HCC.
(A) Correlation between BEX4 expression and tumor grade in HCC patients.
(B) Correlation between BEX4 expression and disease stage in HCC patients.
(C) Correlation between immune checkpoint molecules and BEX4 expression in HCC patients.
(D) Correlation between BEX4 expression and small molecule drug sensitivity.

Figure S5. Validation of BEX4 in GSE64041 dataset.
(A) Comparison of BEX4 expression between paired normal and tumor samples in GSE64041.
(B) Expression correlation between BEX4 and CD20 (B cell marker).
(C) Expression correlation between BEX4 and CD4 (CD4 T cell marker).
(D) Expression correlation between BEX4 and CD8A (CD8 T cell marker).
(E) Expression correlation between BEX4 and CD68 (Macrophage marker).
(F) Expression correlation between BEX4 and CD1C (Dendritic cell marker).
(G) Expression correlation between BEX4 and CD16 (Neutrophil marker).
(H) Expression correlation between BEX4 and TIM3.
(I) Expression correlation between BEX4 and LGALS9.
(J) Expression correlation between BEX4 and PD-L1.

Figure S1

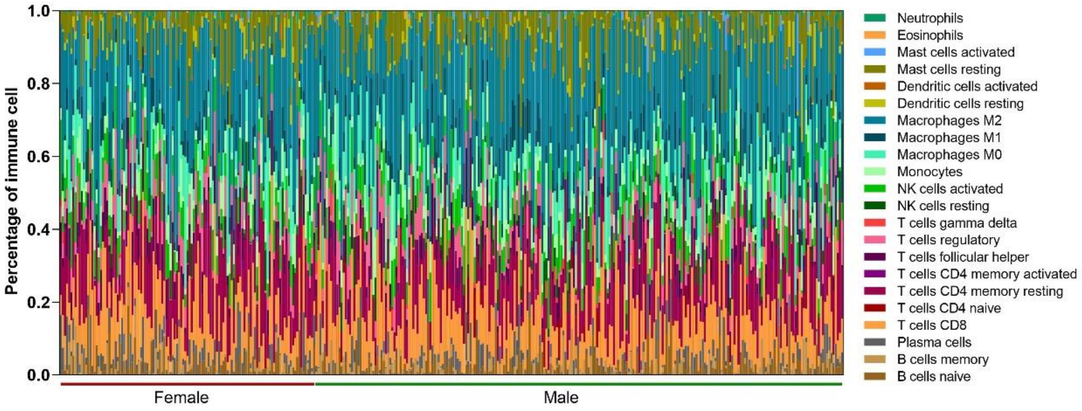

Percentage of immune cell
1.0
0.8
0.6
0.4
0.2
0.0
Female
Male
Neutrophils
Eosinophils
Mast cells activated
Mast cells resting
Dendritic cells activated
Dendritic cells resting
Macrophages M2
Macrophages M1
Macrophages M0
Monocytes
NK cells activated
NK cells resting
T cells gamma delta
T cells regulatory
T cells follicular helper
T cells CD4 memory activated
T cells CD4 memory resting
T cells CD4 naive
T cells CD8
Plasma cells
B cells memory
B cells naive

Figure S2

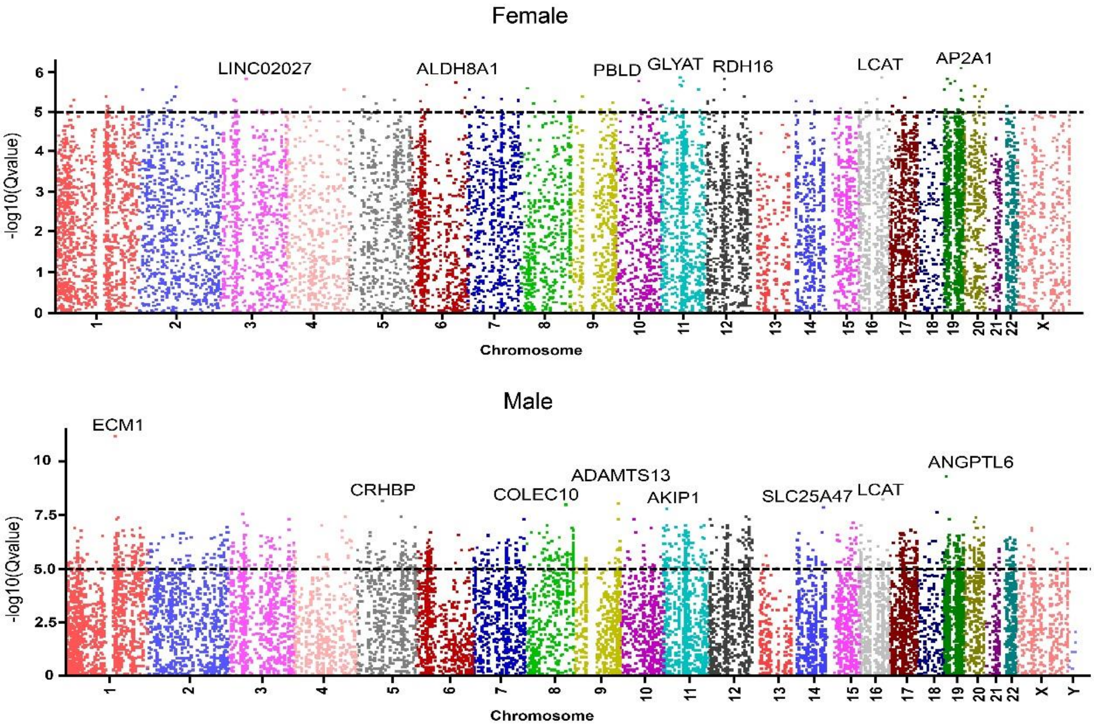
Female
LINC02027
ALDH8A1
PBLD
GLYAT
RDH16
LCAT
AP2A1
-log10(Qvalue)
Chromosome
Male
ECM1
CRHBP
COLEC10
ADAMTS13
AKIP1
SLC25A47
LCAT
ANGPTL6
-log10(Qvalue)
Chromosome

Figure S3

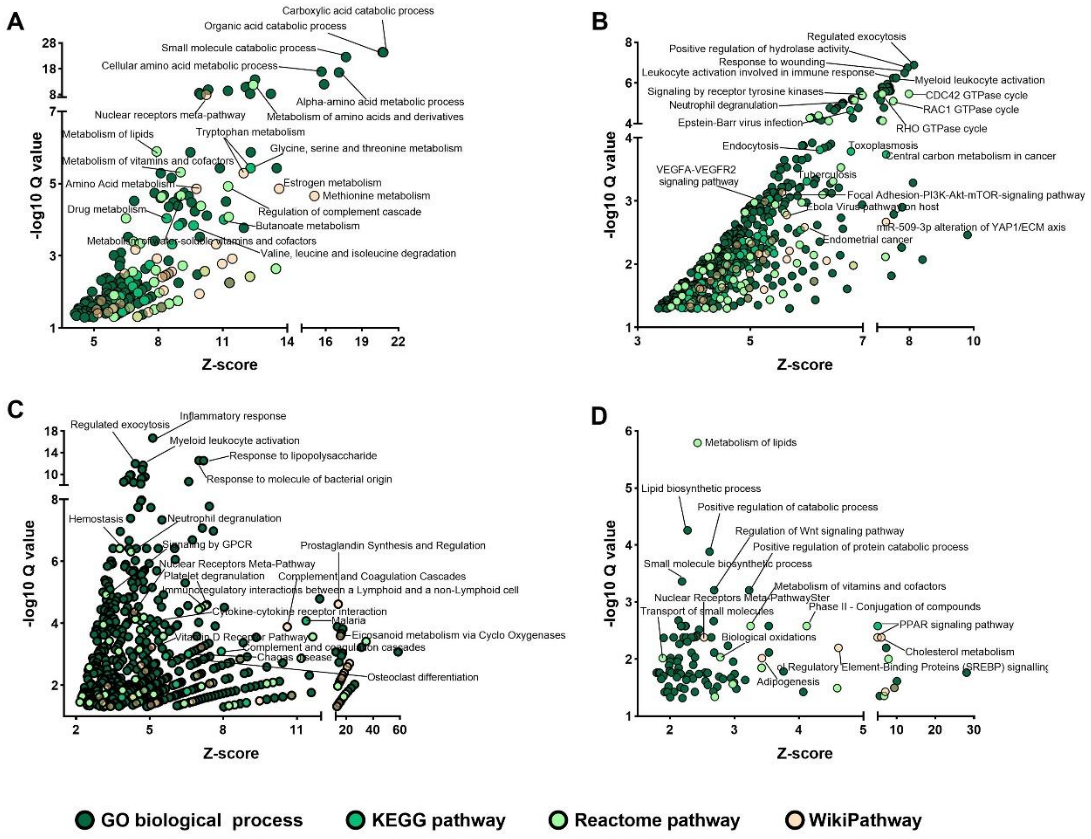
A
Carboxylic acid catabolic process
Organic acid catabolic process
Small molecule catabolic process
Cellular amino acid metabolic process
Alpha-amino acid metabolic process
Metabolism of amino acids and derivatives
Nuclear receptors meta-pathway
Metabolism of lipids
Tryptophan metabolism
Glycine, serine and threonine metabolism
Metabolism of vitamins and cofactors
Amino Acid metabolism
Estrogen metabolism
Methionine metabolism
Drug metabolism
Regulation of complement cascade
Butanoate metabolism
Valine, leucine and isoleucine degradation
-log10 Q value
Z-score
B
Regulated exocytosis
Positive regulation of hydrolase activity
Response to wounding
Leukocyte activation involved in immune response
Myeloid leukocyte activation
Signaling by receptor tyrosine kinases
CDC42 GTPase cycle
Neutrophil degranulation
RAC1 GTPase cycle
Epstein-Barr virus infection
RHO GTPase cycle
Endocytosis
Toxoplasmosis
Central carbon metabolism in cancer
VEGFA-VEGFR2 signaling pathway
Tuberculosis
Focal Adhesion-PI3K-Akt-mTOR-signaling pathway
Ebola Virus pathway on host
miR-509-3p alteration of YAP1/ECM axis
Endometrial cancer
-log10 Q value
Z-score
C
Regulated exocytosis
Inflammatory response
Myeloid leukocyte activation
Response to lipopolysaccharide
Response to molecule of bacterial origin
Hemostasis
Neutrophil degranulation
Signaling by GPCR
Prostaglandin Synthesis and Regulation
Nuclear Receptors Meta-Pathway
Platelet degranulation
Complement and Coagulation Cascades
Immunoregulatory interactions between a Lymphoid and a non-Lymphoid cell
Cytokine-cytokine receptor interaction
Malaria
Vitamin D Receptor Pathway
Eicosanoid metabolism via Cyclo Oxygenases
Complement and coagulation cascades
Chagas disease
Osteoclast differentiation
-log10 Q value
Z-score
D
Metabolism of lipids
Lipid biosynthetic process
Positive regulation of catabolic process
Regulation of Wnt signaling pathway
Positive regulation of protein catabolic process
Small molecule biosynthetic process
Metabolism of vitamins and cofactors
Nuclear Receptors Meta-PathwaySter
Phase II - Conjugation of compounds
Transport of small molecules
PPAR signaling pathway
Biological oxidations
Cholesterol metabolism
Adipogenesis
-log10 Q value
Z-score
GO biological process
KEGG pathway
Reactome pathway
WikiPathway

Figure S4

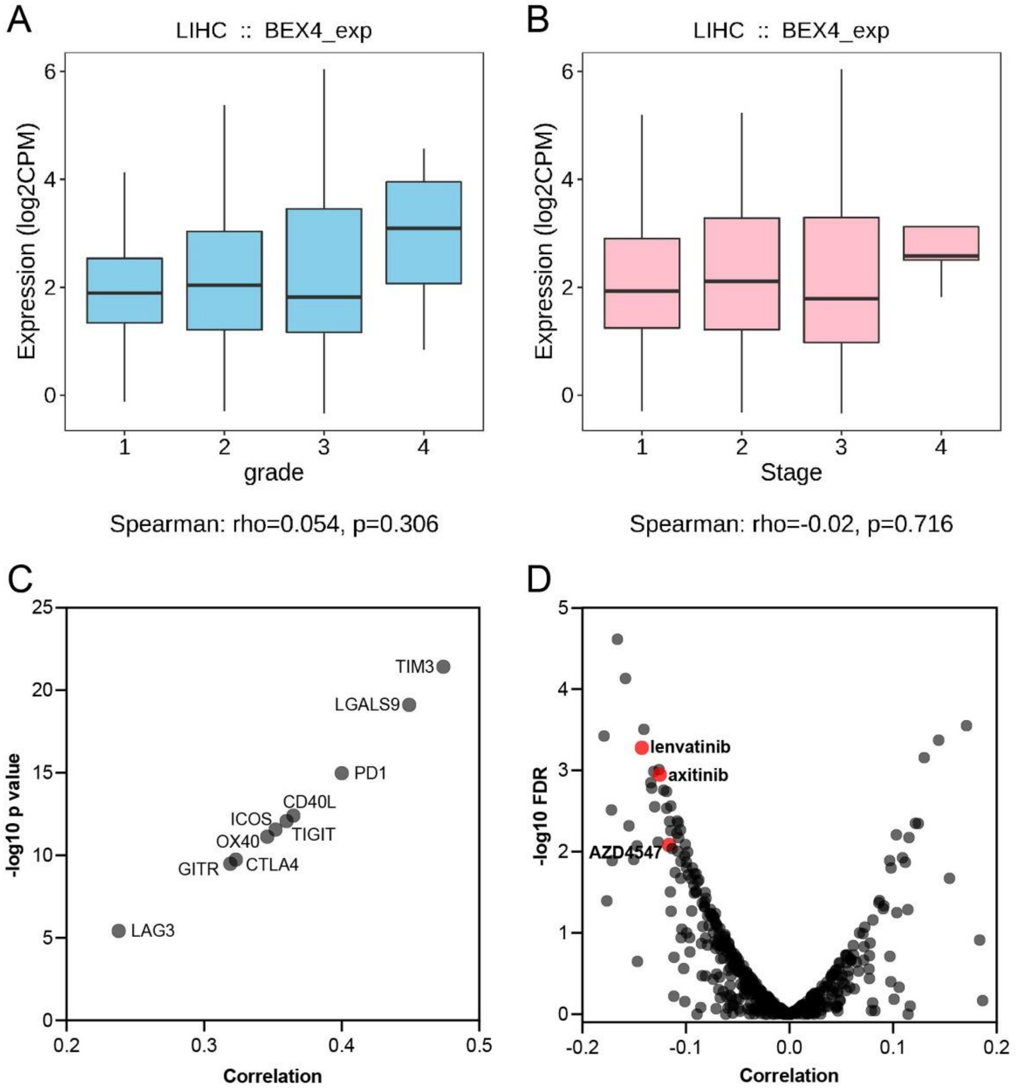
A
LIHC :: BEX4_exp
Expression (log2CPM)
grade
Spearman: rho=0.054, p=0.306
B
LIHC :: BEX4_exp
Expression (log2CPM)
Stage
Spearman: rho=-0.02, p=0.716
C
-log10 p value
Correlation
TIM3
LGALS9
PD1
CD40L
ICOS
TIGIT
OX40
GITR
CTLA4
LAG3
D
-log10 FDR
Correlation
lenvatinib
axitinib
AZD4547

Figure S5

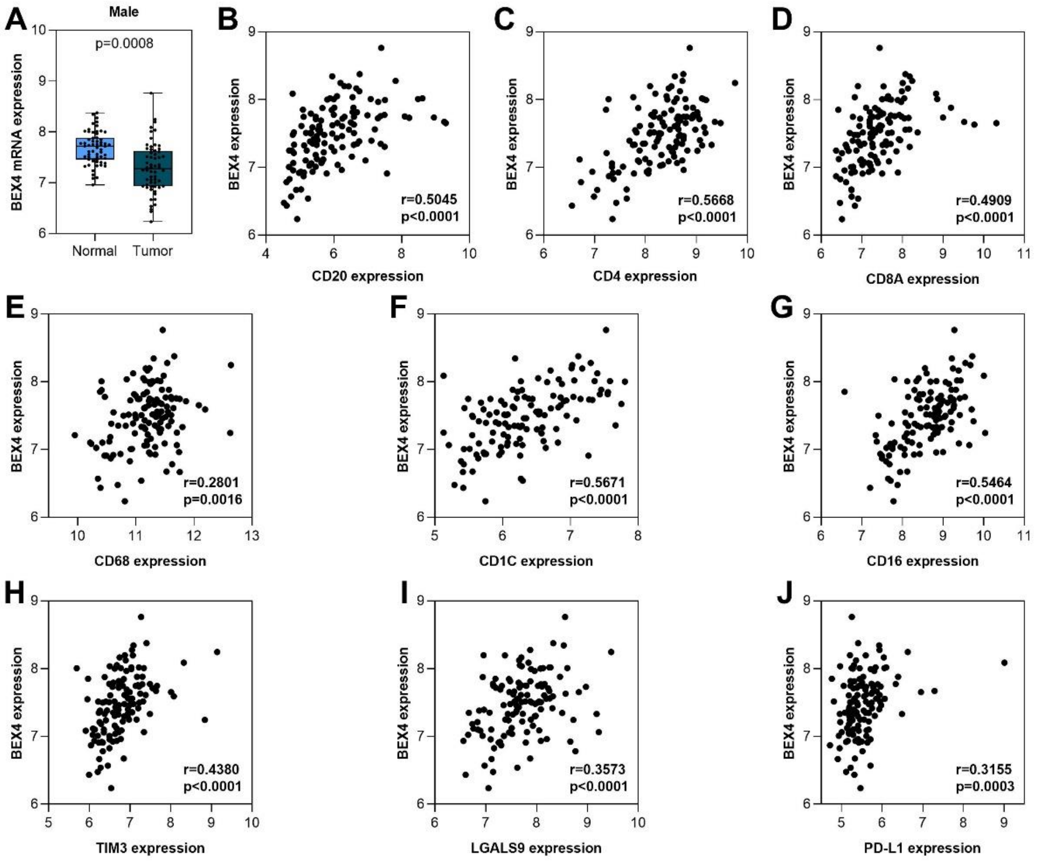

A
Male
p=0.0008
BEX4 mRNA expression
Normal
Tumor
B
BEX4 expression
r=0.5045
p<0.0001
CD20 expression
C
r=0.5668
p<0.0001
CD4 expression
D
r=0.4909
p<0.0001
CD8A expression
E
r=0.2801
p=0.0016
CD68 expression
F
r=0.5671
p<0.0001
CD1C expression
G
r=0.5464
p<0.0001
CD16 expression
H
r=0.4380
p<0.0001
TIM3 expression
I
r=0.3573
p<0.0001
LGALS9 expression
J
r=0.3155
p=0.0003
PD-L1 expression

Table S1. Clinicopathologic characteristics of TCGA cohort

Characteristics	Number of cases (%)
**Age (y)**	
<60	171 (46.1)
≥60	200 (53.9)
**Gender**	
Male	250 (67.4)
Female	121 (32.6)
**Race**	
Caucasian	181 (48.8)
Asian	161 (43.4)
African-American	19 (5.1)
NA	10 (2.7)
**Clinical stage**	
I	175 (47.2)
II	82 (22.1)
III	85 (22.9)
IV	5 (1.3)
NA	24 (6.5)
**Tumor grade**	
G1	54 (14.6)
G2	175 (47.2)
G3	124 (33.4)
G4	13 (4.8)
NA	5 (1.3)
**T classification**	
T1	185 (49.9)
T2	90 (24.3)
T3	80 (21.6)
T4	13 (3.5)
Tx	1 (0.8)
**N classification**	
N0	255 (68.7)
N1	4 (1.1)
Nx	112 (30.2)
**M classification**	
M0	268 (72.2)
M1	4 (1.1)
Mx	99 (26.7)

Table S2. Correlation between BEX4 expression and clinicopathologic characteristics of TCGA cohort

Characteristics	BEX4		P value
**Age (y)**	Low, no. cases	High, no. cases	
<60	78	93	0.1299
≥60	107	93	
**Gender**			
Male	129	121	0.3368
Female	56	65	
**Race**			
Caucasian	94	87	0.521
Asian	74	87	
African-American	10	9	
**Clinical stage**			
I	92	83	0.4089
II	36	46	
III+IV	43	47	
**Tumor grade**			
G1	22	32	0.4528
G2	89	86	
G3	64	60	
G4	5	8	
**T classification**			
T1	95	90	0.6109
T2	39	51	
T3	41	39	
T4	7	6	
**N classification**			
N0	123	132	0.6234
N1	1	3	
**M classification**			
M0	127	141	0.3514
M1	3	1	
